# My Health Too: Investigating the Feasibility and the Acceptability of an Internet-Based Cognitive-Behavioral Therapy Program Developed for Healthcare Workers

**DOI:** 10.3389/fpsyg.2021.760678

**Published:** 2021-12-03

**Authors:** Raven Bureau, Doha Bemmouna, Clara Gitahy Falcao Faria, Anne-Aline Catteau Goethals, Floriane Douhet, Amaury C. Mengin, Aurélie Fritsch, Anna Zinetti Bertschy, Isabelle Frey, Luisa Weiner

**Affiliations:** ^1^Hôpitaux Universitaires de Strasbourg, Strasbourg, France; ^2^Department of Psychology, Laboratoire de Psychologie des Cognitions, Université de Strasbourg, Strasbourg, France

**Keywords:** COVID-19, telehealth, frontline workers, cbt, stress

## Abstract

**Background:** The COVID-19 crisis has had a considerable mental health impact on healthcare workers. High levels of psychological distress are expected to have a significant impact on healthcare systems, warranting the need for evidence-based psychological interventions targeting stress and fostering resilience in this population. Online cognitive behavioral therapy (CBT) has proved to be effective in targeting stress and promoting resilience. However, online CBT programs targeting stress in healthcare workers are lacking.

**Objective:** The aim of our study is to evaluate the feasibility and acceptability of an internet-based CBT intervention, the *My Health Too* program we developed during the first COVID-19 epidemic peak in France.

**Methods:** We recruited 10 participants among Alsace region hospital staff during the first peak of the pandemic in France. They were given 1 week to test the website and were then asked to answer an internet survey and a semi-structured phone interview.

**Results:** We conducted a thematic analysis of the content from the phone interviews. Major themes were identified, discussed and coded: the technical aspects, the content of the website and its impact on participants’ emotions and everyday life. Overall, the participants reported finding the website easy to use and interactive. They described the resources as easy to understand, readily usable, and useful in inducing calm and in helping them practice self-compassion.

**Conclusion:** Our results suggest that the *My Health Too* online CBT program is highly feasible and acceptable to healthcare workers during the highly stressful times of the pandemic peak. The feedback provided helped to improve the program whose efficacy is to be tested.

## Introduction

Healthcare workers are frequently exposed to job stressors (e.g., workload, shift work, patients’ and relatives’ enquiries, patients’ suffering, and team collaboration problems) which have been identified as risk factors for burnout, sleep disturbances and depression (Andela et al., 2016; Dong et al., 2017). The coronavirus disease 2019 (COVID-19) has significantly increased the amount of stressors on healthcare workers worldwide as the number of people infected increased and the disease reached a pandemic status (e.g., lack of beds and equipment, risk of contamination, and a significant number of deaths including the death of coworkers) (Hall, 2020).

A number of psychological consequences of COVID-19 have been reported in the general population and among healthcare workers during the epidemic peak in China and in several other countries (Bohlken et al., 2020; Di Tella et al., 2020; Lu et al., 2020). For instance, the meta-analysis of Liu et al. (2020) found depression and anxiety rates reaching 28 and 33%, respectively in the Chinese general population. In addition, the same authors reported high prevalence rates of depression (50.7%), anxiety (44.7%), insomnia (36.1%), and stress-related symptoms (73.4%) (Liu et al., 2020) among Chinese healthcare professionals, especially nurses (Huang et al., 2020; Mo et al., 2020). Similar findings were subsequently reported in other countries [e.g., France (Horn et al., 2021), Italy (Lasalvia et al., 2020), Indonesia (Setiawati et al., 2021), Turkey (Şahin and Kulakaç, 2021), United States (Forrest et al., 2021)], highlighting an increase in anxiety, depression, and post-traumatic stress disorder (PTSD) symptoms among healthcare workers. A meta-analysis including 38 studies on mental health problems among healthcare workers during the pandemic has reported high prevalence rates for PTSD (49%), anxiety (40%), depression (37%), and distress (37%); these mental health issues particularly affected those working directly with COVID-19 patients (Saragih et al., 2021). Indeed, front line healthcare workers are exposed to more stressors such as increased workload, elevated exposure to deaths and severe cases, risk of contamination and unpredictable working conditions (Batra et al., 2020; Sun et al., 2021), which increase their risk of experiencing distress and burnout (Jalili et al., 2021).

It is clear that the impact of the COVID-19 crisis on mental health, particularly that of healthcare professionals, must be acknowledged. To this aim, recent research has highlighted the importance of mental health prevention measures, with a focus on stress management programs designed for healthcare workers (Hu and Huang, 2020; Maben and Bridges, 2020; Xiang et al., 2020). For instance, phone hotlines for healthcare workers have been set up in a number of hospitals (e.g., Feinstein et al., 2020; Geoffroy et al., 2020; Lissoni et al., 2020; Ravindran et al., 2020; Rolling et al., 2021). However, while these programs provide relief to their immediate distress, they do not sufficiently target those who are developing full-blown stress-induced disorders. Hence, several authors highlighted the need for more tailored support (Ravindran et al., 2020; Wang et al., 2020); particularly interventions based on cognitive-behavioral therapy (CBT; Benhamou and Piedra, 2020; Søvold et al., 2021). Since the beginning of the COVID-19 pandemic, some tailored interventions targeting stress relief have been developed (e.g., Lissoni et al., 2020; Mellins et al., 2020). In Italy, for example, Lissoni et al. (2020) developed a face-to-face intervention aiming to promote resilience among healthcare workers. However, traditional face-to-face psychological therapy has been associated with additional stress in healthcare workers, due to the fear of contracting the virus and the potential interference of the therapy sessions with work schedules (Chen et al., 2020; Lai et al., 2020; Weiner et al., 2020).

Internet-based psychotherapy might be an adequate solution to meet the specific needs of healthcare workers in the context of COVID-19. Indeed, it has the advantage of being accessible to a larger number of people and adapted to the specific schedules of healthcare workers. Specifically, internet-based CBT has proven its effectiveness in the prevention of a number of disorders such as depression (Hagatun et al., 2018), PTSD (Spence et al., 2011), insomnia (Zachariae et al., 2016; Hagatun et al., 2018) and anxiety (Mewton et al., 2014; Hagatun et al., 2018). There is preliminary evidence supporting the effectiveness of internet-based CBT interventions to reduce anxiety and depression in COVID-19 patient samples (Wei et al., 2020), in the general population (Aminoff et al., 2021), and in patients with post-traumatic symptoms related to quarantine or disease (Perri et al., 2021). Similar studies evaluating internet-based CBT interventions are ongoing (Alavi et al., 2020; Bäuerle et al., 2020; Brog et al., 2021). Regarding healthcare workers, data on the effectiveness of internet-based CBT interventions are lacking (Yoo, 2021), particularly in the context of the COVID-19 pandemic. However, prior to the pandemic, internet-based CBT programs were reported to be useful (Guille et al., 2015; Barrett and Stewart, 2021), and preliminary findings suggest that unguided internet-based CBT program for healthcare workers on disability leave might be effective (Miki et al., 2021). In response to the COVID-19 pandemic, similar programs were developed and are currently being evaluated [e.g., mobile-based intervention for frontline healthcare workers by Serrano-Ripoll et al. (2021), internet-based machine-guided stress management program for the general working population by Kawakami et al. (2021)]. Overall, to our knowledge, internet-based CBT interventions targeting stress reduction specifically tailored for the healthcare workers (frontline or not) in the context of COVID-19 are lacking.

To address this unmet need, we developed an open-access CBT-based unguided internet program “*My Health Too*” (in French “*Ma Santé Aussi*”), which aimed at promoting resilience and reducing stress in healthcare workers (Weiner et al., 2020). The aim of our study is to evaluate the feasibility of the beta-version of the program during the first peak of the epidemic in France (Spring 2020). Indeed, it is strongly recommended to conduct feasibility studies to verify whether the intervention is implementable, identify the optimal settings for its implementation and determine whether it deserves further evaluation by a full-scale efficacy study, e.g., randomized controlled trial (Bowen et al., 2009; Tickle-Degnen, 2013; Blatch-Jones et al., 2018). To assess feasibility and acceptability, we were interested in (i) satisfaction indexes (e.g., number of videos viewed, evaluation of the quality and the videos’ duration) collected through an internet survey, as well as (ii) participants’ subjective experience of the intervention collected through individual interviews.

## Materials and Methods

### Participants

Participants included in this preliminary study (*n* = 10) were a convenience sample of healthcare workers. Eligibility criteria included being directly impacted by the COVID-19 healthcare crisis either by working in (or being reassigned to) a unit treating COVID-19 patients or being requisitioned in relation to the crisis. Precisely, three of them were assigned to unfamiliar clinical departments created for people in critical health condition after having contracted the virus, others (*n* = 3) were reassigned to live-in facilities to help with quarantine measures and care for people contaminated with the virus. One was requisitioned, among other duties, to a phone line service for people with autism spectrum disorders who were affected by the quarantine measures. Professions included registered nurses (*n* = 3), special needs educators (*n* = 2), a secretary, a social worker, a paramedic, a medical resident and a nursing student. All participants but one were female (*n*_female_ = 9; *n*_male_ = 1) with ages ranging from 19 to 55 (*M* = 37,8, sd = 11,75) (See Table 1). They were informed about the effort to create curated content and agreed to participate in a beta-test.

**TABLE 1:** Participants demographic table.

**Gender**						
	Male	Female				
	*n* = 1	*n* = 9				

**Age**						
	Min: 19; Max: 55	Mean: 37,8	SD: 11,75			

**Workplace**						
	Hôpitaux Universitaires de Strasbourg	Centre Hospitalier de Rouffach				
	*n* = 8	*n* = 2				

**Profession**						
	Registered nurses	Practicing students	Special needs educator	Paramedic	Social worker	Admin. position
	*n* = 3	*n* = 2	*n* = 2	*n* = 1	*n* = 1	*n* = 1

### Beta Version

The beta version of the *My Health too* website was developed by five clinical psychologists of the East region of France, during the first peak of the epidemic. They created the therapeutic content in collaboration with the *Hackathon* (March 2020^1^) – an event which helps science and health innovators organize around projects – who were responsible for the website layout and technical support. In order to address the acute stress healthcare workers were undergoing, the content was conceived to be readily available through an intuitive and easy-to-use website design. Specifically, the main screen was designed with five smooth colored bubbles. Four of them allowed the users to directly target specific themes the participants could access: “*Understanding stress,”* “*Finding calm,”* “*Sleeping better,”* and “*Staying focused”* (Table 2). The fifth and supplementary icon provided a direct access to all specific available resources. The video and audio documents dispatched to each of the four thematic bubbles targeted the following components identified as key to increasing resilience to stress and preventing mental health problems (Joyce et al., 2014, 2018): (i) psychoeducation (Joyce et al., 2018) (ii) behavioral and cognitive coping strategies (Lazarus and Folkman, 1984), (iii) mindfulness, (iv) mindfulness/acceptance (Hayes et al., 2006), (v) promoting action toward values (Hayes et al., 2006), (vi) addressing barriers and motivation to use self-compassion as a psychological skill (Gilbert, 2014), and (vii) self-compassion to soothe difficult emotion (Neff and Germer, 2013; Gilbert, 2014; Weiner et al., 2020). In total, there were 4 psychoeducational videos of 20 min on average – corresponding to each of the 4 themes available – and 18 audio relaxation and mindfulness exercises whose length ranged between 3 and 28 min. Each item was presented with a title, a small description and the duration of the exercise. The participants were guided throughout their exploration by one of the content creators, who recorded the psychoeducational videos as if she was directly talking to them, encouraging them to take notes or write down what applied to them personally.

**TABLE 2:** Detail of the content.

	Session	Duration	Goal	Content/example strategies	Additional material
(1)	Understanding stress	23′23	Understand the biological and psychological mechanisms of stress	Psycho-education on stress mechanisms	6 mindfulness audios (e.g., building a safe place in imagination, eating fruit in mindfulness, visualizing a compassionate being)
(2)	Finding calm	26′55	Learn strategies to deal with stress in the moment	Taking a break, self-soothing, cognitive distraction, making sense of the situation	3 mindfulness audios (breathing in mindfulness, mindful breathing with words, observing one’s mood)
					2 relaxation audios (muscle relaxation and rhythmic breathing)
					1 audio on problem solving
(3)	Staying focused	16′49	Learn to use mindfulness in everyday life to better cope with stress	Focusing attention deliberately on elements of the present, noticing thoughts, emotions and sensations	6 mindfulness audios (e.g., breathing in mindfulness, taking a break, self-compassion mindfulness)
(4)	Sleeping better	11′15	Discover the main CBTi strategies to improve sleep quality	Psycho-education on sleep and strategies such as disconnecting from work stress using mindfulness, sleep restriction, and stimulus control.	3 mindfulness audios (visualizing a safe place, breathing in mindfulness and end-of-day mindfulness)
					2 relaxation audios (muscle relaxation and rhythmic breathing)
					1 written document with strategies to improve sleep

### Materials and Procedure

Participants were recruited among the staff of the University Hospital of Strasbourg and the Hospital of Rouffach after receiving written information *via* e-mail on the aims of the study. They were then asked to browse the website, watch the videos and use the different audio recordings for a full week. After this period of time, they were invited to give their feedback on their experience, first *via* an internet survey and then through a 1-h semi-structured phone interview. The whole study period ran from May 15th to June 2nd, 2020, a time during which the acute crisis was very slowly beginning to transition to manageable levels. The study was approved by the relevant local ethics committee (Comité de Protection des Personnes Ile-de-France VI, May 7, 2020, N 36-20).

To determine whether the feasibility of our intervention was satisfactory, we determined a progression criterion (Mbuagbaw et al., 2019) which consisted of at least 70% of the participants who completed the assessments (5 out of 7) being able to watch at least two thirds of the program (3 videos) and being overall satisfied with the website. A participant was considered satisfied if he/she answered “agree” to at least 3 of the question 2 items in the section “Introduction” of the online survey.

#### Internet Survey

Prior to the phone interview, participants were asked to answer a short internet survey (17 items, see Supplementary Table). Questions included the type of device used to access the website, how much content they had used and/or viewed and their preliminary feedback about the experience, such as their evaluation on the duration of the videos and exercises.

#### Phone Interviews

Individual phone interviews were conducted by three clinical psychology graduate students trained in the *My Health Too* CBT beta-version program. Participants were asked to give feedback and suggestions regarding their experience with the program as well as potential changes that could improve it.

### Content Analysis

Thematic analyses following the 6-step methodology of Braun and Clarke (2006) were conducted on the qualitative data. This approach is data-driven and allows for alternative perspectives not initially expected by the researchers. Audio recordings were transcribed, and the interview content was coded. Themes were then identified and discussed. Although the number of participants is small, theme saturation was reached, which allowed for data analysis. A consensus was then reached among the three psychology graduate students as to what the major themes were. A global supervision of the process was provided by three senior clinical psychologists (LW, IF, AZ-B).

## Results

### Internet Survey

Seven participants answered the internet survey. Missing responses were mostly due to lack of time as participants were still heavily engaged in crisis management at work. The results presented here include responses received after one reminder (*n* = 7). The participants indicated that they found the website useful, simple to use and interesting (*n* = 7/7). This result fulfils our progression criterion as 100% (≥70%) of participants agreed with at least three of the selected satisfaction criteria. In addition, participants reported that they used the website on their off-days exclusively (*n* = 3/7) or a little everyday (*n* = 3/7) with one participant (*n* = 1/7) splitting their test of the website in two to three evenings.

#### Psychoeducational Videos

Most of the participants (*n* = 4/7) watched all of the psychoeducational videos, with others watching at least 3 (*n* = 2/7) or 2 (*n* = 1/7). This result also fulfils our progression criterion as 86% (≥70%) of participants watched at least 3 videos out of 4. They found the content interesting (*n* = 7/7) useful (*n* = 6/7) and not overly complex (*n* = 6/7). However, the majority (*n* = 5/7) indicated that they found the videos too long while the remainder (*n* = 2/7) found the duration well-balanced.

#### Resources and Exercises

Due to the time constraint of this test, out of the 18 exercises available, the participants only had the time to test on average 6 exercises (*n* = 5/7) with one (*n* = 1/7) being able to try out 6–12 resources and another one (*n* = 1/7) being able to test between 13 and 18 exercises.

All participants (*n* = 7/7) found the exercises useful and easy to understand. They also rather agree (*n* = 3/7) or completely agree (*n* = 4/7) that they were easy to use. The majority of participants found the duration well-balanced (*n* = 5/7) while the remaining two found them a little too long.

All respondents (*n* = 7/7) indicated that they were interested in using this type of resource if another crisis situation were to emerge.

### Phone Interviews

#### Major Themes

Three major themes were identified: technical aspects (technical difficulties, feedback on the user interface, and on the technical aspects of the resources), user feedback on the content of the resources and the impact of the resources on the participants’ lives.

#### Technical Aspects

Regarding the user interface, feedback was highly positive with descriptors such as “*easy*,” “*intuitive*,” “*simple*,” “*interactive*” and “*self-explanatory*.” Some users also described the aesthetic as “*peaceful*,” “*calming*” and “*not too stimulating*” (thus conducive to relaxation). Many also appreciated the flexibility in accessing the content, being able to explore the resources and try the different exercises when they had time to do so in their busy schedules.

However, participants encountered some technical difficulties, depending on the platform they used to access the website (i.e., mobile phone, tablet, or computer). These difficulties were mainly related to the size and placement of some of the texts, links, and information, with some difficulties related to loading times.

Suggestions included the possibility of having a personal account, so they could easily access their favorite resources and visualize their progression throughout the program. Most participants also mentioned that the psychoeducational videos could be presented in smaller increments, following the chapters depicted in the graphics of the videos.

#### Content

Participants used the resources in different ways. Some had access to the website at work during breaks or during their train commute. Most reported using the website at home, “*once everything (work, household chores, childcare, etc.) is over*.” Regarding the criteria that guided their theme choice, one user reported doing it “*academically,”* that is, going through each resource in order of presentation. Most reported their selection being guided by how they felt at the time and sometimes simply by “*curiosity*,” when they felt “*intrigued*” by the title or the description provided for a specific audio recording.

Their choice was also guided by the duration of the content, since a few people were still reeling from the effects of the crisis even though their current schedules had lightened up. It was clear during some phone interviews that now that the non-stop emergencies were less present, the participants had more time to reflect on what had happened and on the impact the crisis had on their lives, both professionally and personally. Some of them mentioned favoring shorter contents, sometimes aligning different ones in a row, rather than longer recordings because “*telling myself “I am going to take 25 min now to watch or listen to this” seems impossible*.”

Participants reported being satisfied with the way the contents were provided, the exercises were “*easy to understand and to use*.” However, the quality of the recordings (e.g., being able to hear some background noises) bothered some participants.

Suggestions included having text summaries of the psychoeducational videos to refer back to as well as glossaries of some “*technical terms*” used in the videos. One person suggested providing indications of when/where to use some of the recordings, especially for people who are not used to the practice of mindfulness or relaxation.

#### Impact

Using the website was described as very useful to “*understand what is going on*,” “*why I feel that way*,” “*what is happening*,” thus paving the way to feeling more in control. Two of the participants reported using the website for “*myself as well as for my patients. Some exercises were difficult for me, because of my history, who I am, etc., but I think it could be very useful for some of my patients*.” One major theme evoked by participants was self-compassion. They expressed their gratitude to the team because they viewed the content as a way to allow themselves to “*think about myself*,” “*not forget that I deserve to take care of myself as well*.” They described testing the website as an opportunity and moment in which they allowed themselves to disconnect from the stress of work, from the ongoing crisis and instead focusing on themselves and how to regulate their difficult emotions “*while trying not to judge myself*” and “to *not feel guilty*.”

One user described applying what was learned almost immediately during a time of crisis. She had tried a breathing and mindfulness exercise in the evening and found it to be very soothing. The following morning, she was called following numerous emergencies at work and felt stressed by what awaited her. During the commute, she remembered the exercise, did it, and described arriving at work “*calmer, and feeling more capable to face the day*.”

All participants appreciated that most exercises were designed to be done discreetly, describing them as “*readily usable*,” “*easily applicable in my everyday life*, *including at work*” and being “*a little break that I can use whenever I feel the need to*.”

Having access to this type of resource was also very much appreciated, since it was available “*all the time*,” “*at the touch of a button*,” “*not too cumbersome in comparison to finding a mental health professional*” and “*available even when other types of mental health interventions are not, thus better fitting my work schedule*.”

Regarding their perceived stress, participants indicated that they felt “*more relaxed*,” “*calmer*,” with “*immediate effects which lasted the whole day*.” They also reported that these moments were akin to “*breaks”* during which they could *“breathe and put things in perspective*” and feel “*Zen, completely relaxed*… *actually feel good!*.”

## Discussion

The aim of this study was to evaluate the user experience and the pertinence of the *My Health Too* beta version website in healthcare providers. Overall, our results indicate that this type of intervention is highly feasible and acceptable by healthcare workers facing high stress levels; all the users found the website useful and with a pleasant interface. We also found that the program seemed to have a positive effect on their current stress levels and overall well-being. Specifically, the participants stated that using the website reminded them to focus on their self-care and helped them to cope with difficult emotions. The progression criteria set were met (over 70% of participants completed at least two thirds of the videos and were satisfied with the website) suggesting that it would be worth launching a full-scale study to evaluate the efficacy of the program.

To our knowledge, our study is the first to report the feasibility and the acceptability of an internet-based CBT program aiming to reduce perceived stress and increase resilience in healthcare workers. Consistent with a systematic review and meta-analysis by O’Connor et al. (2018), the *My Health Too* website was described as “easy to use,” “simple,” “intuitive” by users. Moreover, all the participants reported that they were interested in using this type of resources in case another crisis situation were to emerge. Healthcare workers are exposed to numerous stressors and stress-related complications (Andela et al., 2016; Dong et al., 2017), even more so during a health crisis such as the COVID-19 (Liu et al., 2020). Our results suggest that internet-based programs such as *My Health Too* could be an acceptable tool to improve self-care in those who are usually trained to care for others.

One of the benefits of self-applied online interventions is that they address mental health needs within a physical distancing context. Indeed, many health care workers are at high risk for COVID-19 exposure and fear contaminating themselves and others (Lai et al., 2020; Weiner et al., 2020). Moreover, the availability of public mental-health professionals is limited and an increase in demand is projected (Dong and Bouey, 2020) thus emphasizing the strategic importance of self-applied interventions. It is noteworthy to highlight the World Health Organization (WHO) acknowledges self-care approaches as a fundamental component of good practice in mental health care (World Health Organization [WHO], 2003). Accordingly, our participants reported being very satisfied with the online format as it allowed them to access the content “at the touch of a button” and as it “was available even when other types of mental-health interventions were not.” Despite their very intense schedules and limited time to test the intervention, they still managed to test and rate the content. This indicates that this intervention is feasible even in a high intensity pandemic context.

While the online-format is highly flexible, it can also be associated with feasibility challenges which are inherent to the format (equipment, technical proficiency, etc.) (Fischer et al., 2020). The technical difficulties encountered by the participants related mostly to size and placement of texts in different platforms (mobile phone, computer, tablet) and were forwarded to the developers so that they could be fixed for upcoming versions. Also, the quality of some recordings bothered the participants. Indeed, these were made during the first lockdown period and with minimal technical means. They will need to be further improved for upcoming versions. Another challenge is related to the fact that participants navigated the website as they wished – in an open-access manner –, sometimes neglecting resources that could have been useful to them. A more structured step-by-step progression could be useful to people not familiar with this type of intervention, as some of our participants mentioned *“not knowing where to start.”* Format changes (e.g., step-by-step progression) could improve the feasibility and the efficacy of internet-based psychological interventions, which have been found to be less efficient than face-to-face psychological interventions in the general population during the COVID-19 pandemic (Fischer et al., 2020). We consider that online interventions should become an integral part of the therapeutic arsenal proposed by mental health care services as they can facilitate access to care in healthcare professionals.

The results found in this study are, in general, consistent with the available literature on the efficacy of online self-guided mindfulness-based interventions (Ivtzan et al., 2016) and CBT (Rose et al., 2013). For instance, a meta-analysis by Blanck et al. (2018) on the efficacy of self-applied mindfulness exercises found significant small to medium effect sizes estimates for reducing symptoms of anxiety and depression. In addition to distress reduction, another meta-analysis by Chu and Mak (2020) found that online mindfulness-based interventions had a moderate effect on meaning in life (Hedge’s *g* = 0.53) and this effect was mediated by decentering, increasing self-awareness and attention to positive experience. While our study did not aim to investigate the efficacy of the intervention, it is worth noting that a similar attitudinal change was found among the participants in our study, as they reported feeling better after using the website and “feeling more capable to face the day” and having a “more non-judgmental stance.”

### Limitations

Given the circumstances, our sample size was small, limiting our ability to generalize our results to all healthcare workers facing this type of situation. Furthermore, participants were referred by people involved in the development of the content, which may have impacted their responses even though they were not interviewed by the developers. The limited time participants had to test the resources also had a significant impact on their overall experience and their responses. Further indicators of feasibility such as recruitment rates, response rates and testing the acceptability of a comparator intervention could also have been added. A randomized control trial of a newer version of the website is underway to help us better define the acceptability and the efficacy of this intervention (Weiner et al., 2020).

## Conclusion

In conclusion, these study findings suggest that the *My Health too* online CBT program developed for healthcare workers is feasible and might be relevant for reducing stress and improving well-being in this population. A randomized controlled trial aiming to assess the efficacy of the modified step-by-step *My Health too* program is currently being conducted (Weiner et al., 2020). Self-guided online interventions have the potential to reduce distress and improve general well-being in health care providers and thus mitigate the mental health burden associated with COVID-19. Furthermore, timely, widespread online intervention could bolster healthcare workers’ resilience and thus prevent potential mental-health related complications in this population (Pappa et al., 2020).

## Data Availability Statement

The raw data supporting the conclusions of this article will be made available by the authors upon request.

## Ethics Statement

The studies involving human participants were reviewed and approved by (Comité de Protection des Personnes Ile-de- France VI, May 7, 2020, N 36-20). The patients/participants provided their written informed consent to participate in this study.

## Author Contributions

RB, A-AG, CGFF, and FD wrote the first draft of the manuscript and analyzed the data. LW, IF, AZB, AF, and DB designed the study. LW, DB, AM, and AZB reviewed the first draft of the article. LW, IF, AZB, RB, A-AG, AF, and FD recruited the patients. All authors have given final approval of the version to be published.

## Conflict of Interest

The authors declare that the research was conducted in the absence of any commercial or financial relationships that could be construed as a potential conflict of interest.

## Publisher’s Note

All claims expressed in this article are solely those of the authors and do not necessarily represent those of their affiliated organizations, or those of the publisher, the editors and the reviewers. Any product that may be evaluated in this article, or claim that may be made by its manufacturer, is not guaranteed or endorsed by the publisher.

## Footnotes

1
http://hackinghealth.camp/covid19/
