# Supplementary material for: My Health Too: Investigating the Feasibility and the Acceptability of an Internet-Based Cognitive-Behavioral Therapy Program Developed for Healthcare Workers
Source: Front Psychol. 2021 Dec 3;12:760678. doi: 10.3389/fpsyg.2021.760678 (PMC8677821; doi:10.3389/fpsyg.2021.760678)
Supplement: Supplementary file 1 [file Table_1.DOCX]

Supplementary Material

**Questions from the internet survey**

|  | Question | Answer type | Possible answers |
| --- | --- | --- | --- |
|  | **Section 1: General questions** |  |  |
| 1 | Which device(s) did you use to access the website? | Multiple choice | Smartphone  Tablet  Computer |
|  |  |  |  |
| 2 | What is your overall opinion of the website?  a. It is useful  b. It is is nice to use  c. It is easy to use  d. It is interesting | Scale of agreement | Disagree  Neither agree nor disagree  Agree a little  Agree  No opinion |
|  |  |  |  |
| 3 | How many videos did you watch? | Single answer | 1 – 2 – 3 – 4 |
|  |  |  |  |
| 4 | How many pratical tools/exercises have you tested? | Single anwer | None  Less than 6  Between 6 and 12  Between 13 and 18  More than 18  All of them |
|  |  |  |  |
| 5 | How did you use the website? | Multiple choice | A little everyday  One « bubble » a day  Only during my off days  In one very intensive sitting |
|  |  |  |  |
|  | **Section 2 : Specific questions about the videos** |  |  |
|  |  |  |  |
| 6 | You found the videos :  a- Interesting  b - Useful | Agreement scale | Disagree  Neither agree nor disagree  Agree a little  Agree  No opinion |
|  |  |  |  |
| 7 | (Understanding) You found the videos | Scale | 5 points from too simple to too complex |
|  |  |  |  |
| 8 | Regarding the duration, you think the videos are : | Single answer | Too long  Too short  Well-balanced |
|  |  |  |  |
| 9 | Did you, at one point :  a. not watch a video until the end ?  b. switch videos without finishing the one you were watching ?  c. watch a video while doing something else ?  d. change videos because you already knew what was presented ? | Single answer | Yes  No  Don’t want to answer |
|  |  |  |  |
|  | **Section 3 : Specific questions about the exercises** |  |  |
|  |  |  |  |
| 10 | You found the practical tools/exercises :  a. useful  b. easy to do  c. easy to understand | Scale of agreement | Disagree  Neither agree nor disagree  Agree a little  Agree  No opinion |
|  |  |  |  |
| 11 | Regarding the duration, you found the exercises : | Single answer | Too long  Too short  Well-balanced |
|  |  |  |  |
| 12 | After using the exercices, you feel : | Scale | 5 points from Less stressed than before to More stressed than before |
|  |  |  |  |
| 13 | Did you, at one point :  a. not listen to an exercise recording until the end ?  b. switch exercise recording without finishing the one you were listening to ?  c. listen to an exercise recording while doing something else ?  d. change exercises because you already knew how it worked ? | Single answer | Yes  No  Don’t want to answer |
|  | **Section 4 : Ending questions** |  |  |
|  |  |  |  |
| 14 | In the future, if you wanted resources like these, would you go to : | Multiple choice | A website like this one  A mental health professionnal  Other : (free text) |
|  |  |  |  |
| 15 | Why ? | Free text |  |
|  |  |  |  |
| 16 | What benefit do you think using the resources brought you? | Free text |  |
|  |  |  |  |
| 17 | Do you want to add something? | Free text |  |
